# Somatic symptom and related disorders in the Arab world: a narrative review of clinical features and care implications

**DOI:** 10.3389/fpsyt.2025.1692267

**Published:** 2025-12-12

**Authors:** Mario Eid, Venise Abi Kheir, Maya Bizri, Amine Larnaout, Samer El Hayek

**Affiliations:** 1 Department of Psychiatry, Lebanese American University, Beirut, Lebanon; 2 University of Balamand Medical School, University of Balamand, El Koura, Lebanon; 3 Department of Psychiatry, American University of Beirut Medical Center, Beirut, Lebanon; 4 Razi Hospital, Faculty of Medicine, University of Tunis El Manar, Tunis, Tunisia; 5 American Center for Psychiatry and Neurology, Dubai, United Arab Emirates

**Keywords:** somatic symptom disorder, illness anxiety, functional neurological symptom disorder, factitious disorder, Arab countries

## Abstract

**Background:**

Somatic symptom and related disorders (SSRDs) are prevalent worldwide, but their expression, help-seeking patterns, and management are strongly shaped by cultural context. In the Arab world, where mental health stigma and distinct explanatory models are common, SSRDs remain under-studied despite their clinical and public health significance.

**Methods:**

We conducted a narrative review of 35 studies published through June 2025 on SSRDs in Arab populations. Searches of PubMed, Scopus, and Google Scholar identified observational studies, case reports, and cross-sectional surveys addressing Diagnostic and Statistical Manual of Mental Disorders, Fifth Edition, Text Revision (DSM-5-TR) SSRD categories.

**Results:**

Somatic symptoms disorder (SSD) was the most frequently investigated disorder, with prevalence ranging from 12% to 46%. Across studies, female gender, low educational attainment, chronic medical comorbidity, and trauma history were consistent risk factors. Common symptoms included muscle, back, and abdominal pain, often accompanied by depression and anxiety. Illness anxiety disorder, functional neurological symptom disorder, and factitious disorders were less studied but carried important diagnostic, cultural, and service delivery challenges.

**Conclusion:**

SSRDs are common but under-recognized in Arab contexts. Effective management requires early detection in primary care, culturally sensitive communication, and disorder-specific interventions. Future research should broaden the scope beyond SSD, examine longitudinal and trauma-related pathways, and evaluate culturally adapted models of care.

## 1 Introduction

Medically unexplained symptoms (MUS) are persistent bodily complaints for which adequate examination does not reveal sufficiently explanatory structural or other specified pathology (1). MUS are common worldwide and occur at particularly high rates in non-Western cultures and among ethnic minorities in high-income countries (2, 3). Their presentation is influenced by sociocultural factors, including linguistic tendencies to convey distress through bodily idioms and culturally shaped health beliefs (4). Distinct regional patterns have been described: for example, African populations may report heat sensations, numbness, and diffuse pain (5); Spanish primary care patients frequently present with backache (6); and patients in Saudi Arabia often report pain and neurological symptoms (7).

The term *somatization* denotes the expression of psychological distress through physical symptoms that lack an adequate medical explanation. In earlier classifications, persistent physical symptoms were grouped under “somatoform disorders”, which were defined as physical symptoms suggesting a physical disorder without demonstrable organic findings and with strong evidence of psychological causation (8). With the publication of the fifth edition of the Diagnostic and Statistical Manual of Mental Disorders (DSM-5), this category was replaced by “Somatic Symptom and Related Disorders” (SSRDs) (9). The text revision DSM-5-TR includes somatic symptom disorder (SSD), illness anxiety disorder, functional neurological symptom disorder (FNSD), factitious disorder, and factitious disorder imposed on another. Disorders previously grouped under somatization, e.g., body dysmorphic disorder and pain disorder, are now classified separately (10). This updated categorization emphasizes the presence of distressing physical symptoms along with disproportionate thoughts, feelings or behaviors, rather than the absence of medical explanation, and underscores the interplay between physical symptoms and mental health (1).

Globally, population surveys indicate that approximately 26–35% of adults attending primary care meet criteria for a somatoform or SSD, and cross-cultural reviews emphasize that somatization is a universal phenomenon, with differences occurring primarily in symptom expression rather than overall prevalence (11, 12). Given their high prevalence, functional impairment, and complex presentation, SSRDs represent a major yet under-recognized public health concern, particularly in regions where cultural factors strongly influence the expression and interpretation of psychological distress.

The Arab region comprises 22 countries with a combined population of approximately 425 million, nearly 60% of whom are under the age of 25 (13). Psychiatric disorders account for an estimated 5.6% of the total disease burden in the region, with depressive disorders contributing the largest share of disability-adjusted life years (14). Suicide is also a growing concern, with 26,000 deaths reported in the Eastern Mediterranean Region in 2016 (15). Despite this burden, mental health service provision remains limited, averaging 7.3 mental health workers and 4.2 psychiatric beds per 100,000 population, compared with 43.5 and 35, respectively, in Europe (16).

In the Arab world, distinct explanatory models of organic and psychiatric illnesses are common (17, 18). Explanatory models encompass the patient and family’s beliefs about illness causes, personal and social meanings, and expected management. These beliefs often incorporate biomedical, psychological, and supernatural elements and shape treatment outcomes (19). In this regard, cultural, religious, and traditional values play a central role in shaping how mental illness is perceived and expressed in Arab societies (20, 21). Socioeconomic challenges, including poverty, gender inequality, stigma, and limited access to specialized care, further influence health-seeking behaviors and the likelihood of receiving appropriate mental health services (22, 23). These sociocultural and structural factors are particularly relevant to SSRDs, as they can affect both the types of symptoms reported and the diagnostic process. For clinicians working in multicultural settings, understanding such cultural dynamics is crucial, because differences in explanatory models, cultural values, and doctor-patient relationship preferences are known to cause communication problems and misinterpretation of symptoms (24).

Although global evidence indicates broadly comparable prevalence rates for SSRDs, reported figures across studies often diverge because of differences in diagnostic criteria, sampling methods, and cultural interpretations of MUS, rather than genuine epidemiological variation (25–29). However, little is known about how these patterns manifest specifically within Arab populations, and no comprehensive review has yet synthesized the available evidence.

The present narrative review addresses this gap by summarizing published research on the prevalence, epidemiological characteristics, and associated factors of SSRDs in Arab countries. It also identifies knowledge gaps and outlines recommendations to guide culturally appropriate clinical practice and policy in the region.

## 2 Material and methods

Given the heterogeneity of diagnostic labels (e.g., somatoform disorder, somatization, bodily distress, etc.), study designs (e.g., case reports, cross-sectional surveys), populations (primary care, hospital outpatients, community samples, refugees, and students) and outcome measures (prevalence, symptom patterns, risk factors, and comorbidities), a formal systematic review or meta-analysis was not feasible. Instead, a narrative review was conducted, providing a comprehensive descriptive synthesis of the diverse data and integrating the relevant cultural context.

A literature search was conducted in PubMed, MEDLINE, Scopus, and Google Scholar from database inception to June 15, 2025. Search terms included "somatic symptom disorder", “somatoform disorder”, “somatization”, "illness anxiety", "hypochondriasis", "conversion disorder", "functional neurological symptom disorder", and "factitious disorder", in combination with "Arab countries" and the names of individual Arab nations. Reference lists of relevant publications were also screened to identify additional studies. No formal language restrictions were imposed.

Eligible studies met the following criteria: (1) conducted in Arab populations, and (2) addressed SSRDs or related diagnoses consistent with DSM-5-TR categories. Observational studies, cross-sectional studies, and case reports were included to capture both clinical trends and culturally specific presentations. For each eligible study, the following information was extracted: study design, country, setting and participant characteristics; diagnostic criteria and screening instruments; sample size and prevalence estimates; symptom patterns; risk factors and comorbidities; management approaches; and authors’ key conclusions. Findings from the included studies were narratively synthesized.

## 3 Results

A total of 35 studies conducted across various Arab countries were identified, each addressing a specific subtype or presentation of SSRDs. Of these, 18 studies examined SSD, seven addressed illness anxiety disorder, four investigated FNSD, five focused on factitious disorder, and one described a culture-bound syndrome (Dhat syndrome).

**Figure 1:**
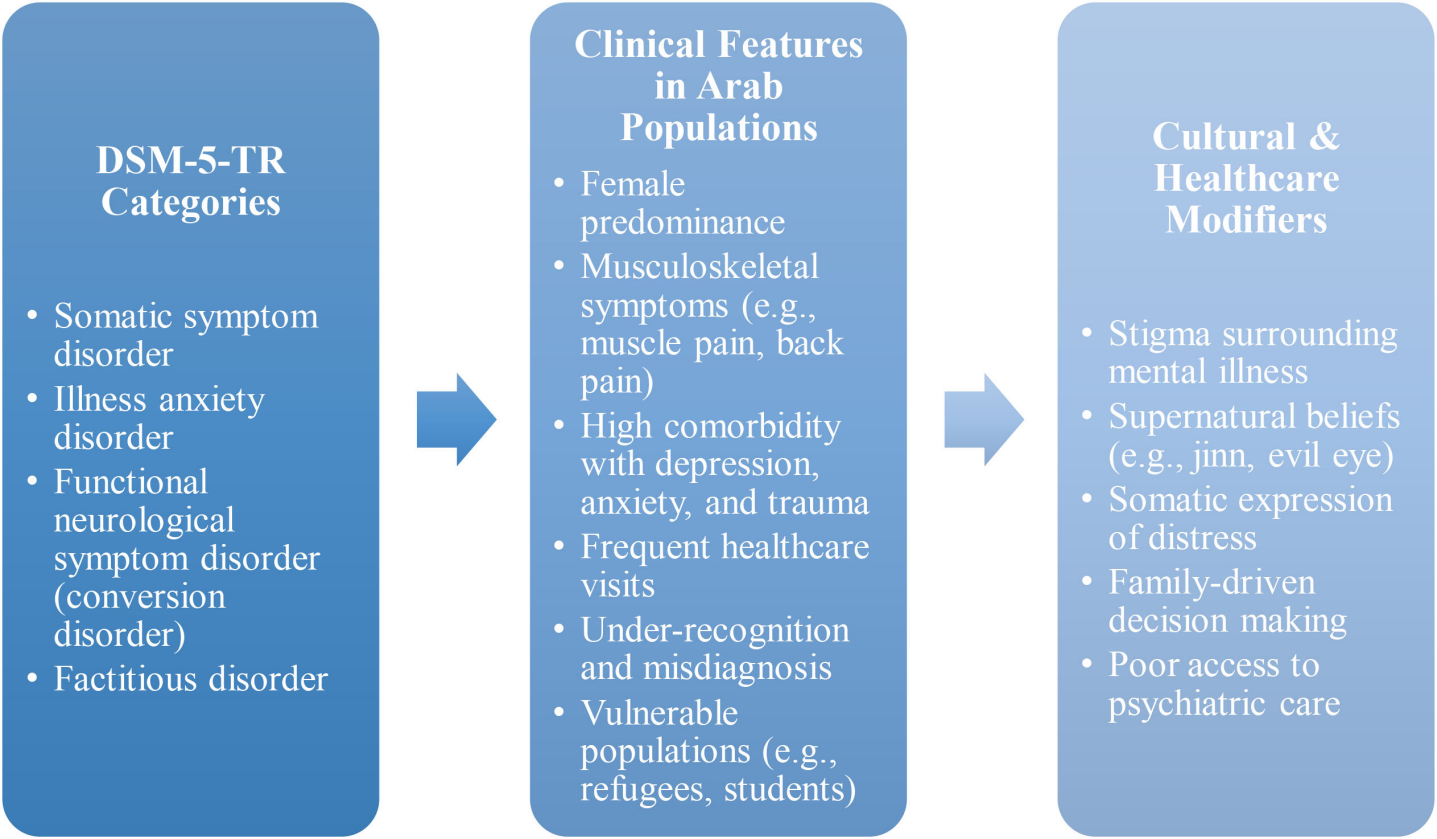
Somatic symptom and related disorders in the Arab World: conceptual framework. Legend: This conceptual framework illustrates how somatic symptom and related disorders (SSRDs), as classified by the Diagnostic and Statistical Manual of Mental Disorders, Fifth Edition, Text Revision (DSM-5-TR), are expressed and influenced within Arab populations. The left layer outlines the primary diagnostic categories under the SSRDs umbrella: somatic symptom disorder, illness anxiety disorder, functional neurological symptom disorder, and factitious disorder. The middle layer presents the core clinical features frequently observed in Arab populations. These include a predominance among females, common symptom patterns (e.g., musculoskeletal symptoms, headaches), high rates of psychiatric comorbidities such as depression, anxiety, and trauma-related symptoms, and frequent general healthcare visits. The right layer identifies key cultural and systemic modifiers that shape how these disorders are experienced, interpreted, and treated. These include stigma surrounding mental illness, religious or supernatural attributions, and limited access to psychiatric services, among others. This model underscores the necessity of using a culturally informed, biopsychosocial approach to diagnosing and managing SSRDs in Arab contexts.

A conceptual framework illustrating the manifestation of SSRDs in Arab populations is presented in **Figure 1**. This framework integrates DSM-5-TR diagnostic categories with common clinical features and sociocultural determinants specific to the region, offering a contextual lens for interpreting the findings described below.

### 3.1 Somatic symptom disorder

#### 3.1.1 Study origins and sample sizes

Studies on SSD originated from eight Arab countries: Bahrain (n=1) (30), Kuwait (n=1) (25), Oman (n=2) (31, 32), Palestine (n=2) (33, 34), Qatar (n=4) (35–38), Saudi Arabia (n=3) (7, 39, 40), Tunisia (n=1) (41), and the United Arab Emirates (UAE) (n=2) (42, 43). Two studies addressed Syrian refugees (n=2) (44, 45).

Sample sizes varied from 38 (31) to 2,000 participants (32). Details of these studies and their main findings are summarized in **Table 1**.

**Table 1:** Summary of studies discussing somatic symptom disorder in the Arab world.

Study	Country	Pooled sample	Scales used	Main findings
Alalawi et al. (32)	Oman	Adult Omani patients attending healthcare centers in Muscat; n=2,000	Somatic Symptom Scale-8	-The prevalence of SSD was 30.1%. -SSD was significantly associated with female gender (p<0.001), high school education (p<0001), and the presence of chronic medical comorbidities (p=0.001).
Abdelaziz et al. (30)	Bahrain	Year 2 and Year 6 medical students at the Arabian Gulf University; n=160	Patient Health Questionnaire (PHQ)-15, PHQ-9, Generalized Anxiety Disorder (GAD)-7	-The prevalence of somatic symptoms was 46%. -Higher levels of somatic symptoms were found in females compared to males (p<0.05). -Year 2 students reported higher somatic symptom levels than year 6, but this was not statistically significant. -Students had notable levels of anxiety (39.4%) and moderate-to-severe depression (18.8%).
Al-Adawi et al. (31)	Oman	Women diagnosed with undifferentiated somatoform disorder and healthy subjects; n=38	Composite International Diagnostic Interview, tests for neurocognitive functioning, Bradford Somatic Inventory	-Significant differences between patients with undifferentiated somatoform disorders and normal healthy subjects were found on tests of working memory and executive functioning (p<0.001), perseverative errors (p<0.001), sleep quality (p<0.001), anxiety (p<0.001), and somatization (Bradford Somatic Inventory 57.0 vs. 27.4, p<0.001).
Alanazi et al. (7)	Saudi Arabia	Patients at King Abdulaziz Medical City; n=89	–	-The most prevalent SSRDs were somatization (77.5%), followed by conversion (13.5%), pain disorder (5.6%), and hypochondriasis (3.4%). -The most common presenting symptoms in the somatization group were pain (56.2%) and neurological symptoms (46.4%). -53.9% initially presented to the outpatient clinic, while the remaining participants attended the emergency department. -41.6% of patients received no pharmacological treatment. When received, treatment mainly included antidepressants (46%), anticonvulsants (16.9%), and atypical antipsychotics (11.2%).
Aleyeidi et al. (39)	Saudi Arabia	Community adults; n=1,377	Somatic Symptom Scale−8, Cyberchondria Severity Scale	-52.6% of participants exhibited high cyberchondria/high somatic symptom scores. -Younger age (p<0.001), female gender (p<0.001), having chronic diseases (p=0.004), lower monthly income (p=0.002), and frequent internet search (p=0.04) were significantly associated with increased severity of cyberchondria and somatic symptoms. -Affordability and the sense of confidence provided by internet self-diagnosis were key factors influencing self-management intentions in 54.6% and 34.1% of participants, respectively.
Alkhadhari et al. (25)	Kuwait	Individuals attending primary care clinics in Kuwait; n=1,046	PHQ-Somatic/ Anxiety/ Depression (PHQ-SAD) (comprises PHQ-9, PHQ15, and GAD-7)	-42.3% and 64.1% had at least one mental condition and a physical illness, respectively, with 34.4% having a mental-physical comorbidity. -Somatization was the most common mental condition comorbid with physical illnesses, occurring in 20.8% of cases. -Somatization score was associated with a higher number of physical illnesses (mean PHQ-9 = 6.8 for no physical illness, 8.5 for one, 9.6 for two, and 11.1 for three or more physical illnesses, p<0.001). -Patients with asthma had significantly higher somatization scores compared to those with diabetes and hypertension (mean PHQ-9 = 11.8, 8.3, and 8.9, respectively, p<0.01).
Alqahtani & Salmon (40)	Saudi Arabia	Patients attending primary care; n=224	12- and 28-item versions of the General Health Questionnaire	-The prevalence of psychological disorders was 43% and 53%, as per the 12- and 28-item versions of the General Health Questionnaire, respectively. -The most prevalent psychological morbidities were depression (27%), anxiety (25%), and somatic symptoms (16%). -The prevalence of somatization was 14%. -Females reported more somatic symptoms than males (p<0.001). -The most common presenting symptom was headache.
Bener, Al-Kazaz, et al. (35)	Qatar	Patients attending primary care; n=1,762	PHQ-15	-The prevalence of somatization, depression, anxiety, and stress was 11.7%, 11.3%, 8.3%, and 18.6%, respectively. -9.3% of participants had comorbid depression, anxiety, somatization, and stress. -The most common symptoms among males and females were stomach pain (64.9% and 65.3%, respectively) and pain in arms/legs/joints (51.4% and 43.1%, respectively). -51.7% and 50% of patients with depression and anxiety, respectively, had somatic symptoms. -50% and 35.4% of patients with somatoform disorders had symptoms of depression and anxiety, respectively.
Bener, Dafeeah, et al. (36)	Qatar	Patients attending primary care; n=1,762	PHQ-15	–The prevalence of somatic symptoms was 13% (229 out of 1,762 subjects). -48.5% had more than four somatic symptoms, and 31.9% had severe symptoms. -Those with somatic symptoms also had depression (15.3%), anxiety (8.7%), and stress disorders (19.2%). -Gender differences were found in age group (p=0.001), nationality (p<0.001), and profession (p<0.001)
Bener, Ghuloum, & Burgut (37)	Qatar	Qatari patients attending primary healthcare centers; n=1,689	12-item General Health Questionnaire, Clinical interview schedule	-The prevalence of somatoform disorders was 23.9%. -The most reported symptoms were backache in men (45.5%) and headache in women (55.4%). -The prevalence of somatoform disorders was slightly higher in females (24.2%) compared to males (23.7%). -There was a significant difference in somatic symptoms between men and women based on their age group (p=0.031) and level of education (p=0.008), but not their marital status. Specifically, men between 30 and 39 years and women between 40 and 49 years had higher occurrences of symptoms. In addition, housewives and men in administrative posts had higher rates of somatoform disorders compared to other jobs. -Physical illnesses during childhood (p=0.022) and adulthood (p=0.029) were significantly higher in men with somatoform disorders. -Psychiatric family history (p=0.012), prolonged depressive reaction (p=0.003), and discontent with social life (p<0.001) were significantly higher in women with somatoform disorders.
Bener, Ghuloum, Al-Mulla, et al. (38)	Qatar	Qatari patients attending primary n=1,689	12-item General Health Questionnaire, Clinical Interview Schedule	-The prevalence of somatization was 12.4%. -Somatization was significantly more frequent among middle-aged patients, particularly those aged 30–39 years (p=0.01). -Predictors of somatization: general health (p<0.001) and adverse childhood experiences (p=0.003). -Most diagnostic categories and the severity of psychiatric illness were higher in patients with psychologization compared to those with somatization. -No significant differences were observed for gender, marital status, education, or profession.
Borho et al. (44)	Syrian refugees	Syrian refugees in Germany; n=116	PHQ-15	-The prevalence of somatic distress risk was 49.1%. -The prevalence of moderate-to-severe distress was 24.1%. -The most distressing symptoms were back pain (22.4%) and pain in arms/legs/joints (17.2%). -Factors significantly associated with somatic distress: female gender, higher number of medical visits, traumatic experiences, more different trauma types, and higher depression and anxiety scores (p<0.05).
El-Rufaie et al. (42)	United Arab Emirates	Patients attending a primary health care center in Al-Ain; n=652	12-item General Health Questionnaire, Clinical Interview Schedule	-The prevalence of somatoform disorders was 12%. -Headache (45%), backache (35%), and abdominal pain (30%) were the most common somatic symptoms. -Other symptoms included pain in the upper and lower limbs (19%), fatigability (19%), generalized aches and pains (16%), chest pain (15%), pain or discomfort in the throat (9%), and breathlessness (9%). -26% presented with one symptom, 29% presented with two symptoms, and 45% presented with three or more symptoms. -The duration of presenting symptoms was 1 year in 23% of patients, 1–2 years in 23%, and >2 years in 48%. -Symptoms were mild in 18% of patients, moderate in 49%, and severe in 34%. -There was no statistically significant difference between somatoform disorders and psychologized mental disorders groups in terms of age, gender, marital status, occupation, and smoking habits. However, the educational level was significantly lower among the former group (p=0.03).
Halak & Idris (33)	Palestine	Visitors to major hospitals in the Hebron Governorate; n=523	Family Risk Factor Scale	-Among participants, 299 had psychosomatic conditions. -Experiences of childhood physical, emotional, and sexual abuse were found to be significant among individuals with psychosomatic disorders. -This was more observed among males, those aged 31–40 years, divorced parents, those married for more than 11 years, those with 4–5 children compared to fewer than 4 or more than 6, and those with less than secondary school education.
Hiar et al. (41)	Tunisia	Tunisians exposed to the Arab Spring events; n=60	PHQ-15	-Peri-traumatic dissociation was independently associated with somatic complaints (p=0.03). -Somatic complaints were independently associated with both 12-month physical and mental quality of life (p=0.019). -Somatic symptoms significantly mediated the relationship between peri-traumatic dissociation and physical and mental quality of life.
Mahmoud et al. (43)	United Arab Emirates	Adults residing in the UAE; n=530	Composite International Diagnostic Interview	-57.2% of participants suffered from at least one mental disorder, with higher prevalence rates in women than in men (p=0.029). -Anxiety (56.4%), depression (31.5%), and post-traumatic stress disorder (15.1%) were the most prevalent disorders. -The prevalence of somatoform disorder was 4%.
McGrath et al. (45)	Syrian refugees	Syrian refugees in Istanbul, Turkey; n=1,678	PHQ-15	-The prevalence of high somatic distress was 41.7%. -The most distressing symptoms were pain in arms/legs/joints (38.4%), back pain (38%), and headaches (28%). -Factors positively associated with somatic distress: female gender (odds ratio OR=2.91, 95%, 95% CI = 2.32-3.63, p<0.001), chronic disease (OR=2.87, 95% CI = 2.28-3.61, p<0.001), depression (OR=3.05, 95% CI = 2.32-4.01, p<0.001), anxiety (OR=4.64, 95% CI = 3.50-6.15, p<0.001), post-traumatic stress disorder (OR=1.75, 95% CI = 1.28-2.39, p<0.001), and having a family member with mental health problems (OR=1.65, 95% CI = 1.30-2.09, p<0.001), - Factors positively associated with somatic distress: living in an average (OR=0.64, CI = 0.52-0.80, p<0.001) or good/very good (OR=0.53, 95% CI = 0.31-0.88, p=0.015) household economic situation, and higher education (OR=0.95, 95% CI = 0.93-0.98, p=0.001).
Nazzal et al. (34)	Palestine	Palestinian primary healthcare attendees; n=400	16-point Somatization Scale of the Four-Dimensional Symptom Questionnaire	-The prevalence of SSD was 32.5%. -Muscle pain was the most common symptom (61.5%), followed by back pain (52.3%) and tingling in the fingers (43.8%). -Factors significantly associated with SSD: female gender (adjusted OR aOR=2.1, 95% CI = 1.2-3.7, p=0.043), chronic diseases (aOR=2.4, 95% CI = 1.3-4.5, p=0.01), high depression scores (aOR=3.3, 95% CI = 2.0-5.5, p<0.001), and high anxiety scores (aOR=2.1, 95% CI = 1.2-3.6, p=0.031). -Affected patients had a higher frequency of doctor visits compared to healthy individuals (aOR=2.4, 95% CI = 1.4-4.1, p=0.003).

#### 3.1.2 Study settings and populations

Most studies were conducted in primary care settings and involved adults attending primary health care clinics (25, 32, 34–38, 40, 42). Other populations included hospital-based patients seeking treatment or follow-up services (7, 33, 43), community-dwelling adults (39, 41), Syrian refugees residing in host countries (44, 45), and medical students (30).

#### 3.1.3 Prevalence

The prevalence of somatic symptoms/somatization ranged from 12% (35, 37, 42) to 46% (30). Conversely, the prevalence of “somatoform disease” ranged from 4% (43) to 32.5% (34).

One study reported high somatic symptom scores in 52.6% of participants (39). Among Syrian refugees, bodily distress was common, ranging from 41.7% (45) to 49.1% (44).

#### 3.1.4 Risk factors

Across studies, female gender was the most frequently reported risk factor (30, 32, 39, 40, 44, 45).

Several studies reported gender-specific patterns: differences in somatic symptoms by age group (35, 37), level of education (38), nationality, and profession (36). Men were more likely to report physical illnesses during both childhood and adulthood, whereas women showed higher rates of psychiatric family history, prolonged depressive reactions, and dissatisfaction with social life (37).

Other significant risk factors included younger age (39), lower education level (32, 42, 45), lower socioeconomic status (39, 45), chronic medical comorbidities (32, 34, 45), positive psychiatric family history (45), and high depression and anxiety scores (34, 44, 45).

Past trauma (44, 45) and trauma-related factors were also prominent. These included childhood abuse, exposure to traumatic events, and emotional neglect (33, 38), with the greatest impact observed in males, individuals aged 31–40 years, those with divorced parents, and those with less than secondary school education (33). In Tunisia, peri-traumatic dissociation was independently associated with somatic complaints one year after the Arab Spring events. Somatic symptoms also mediated the relationship between peri-traumatic dissociation and reduced physical and mental quality of life (41).

#### 3.1.5 Symptomatology

Commonly reported symptoms included pain (7), particularly:

- Muscle and back pain (34, 37, 42, 44, 45),
- Stomach pain (35),
- Aches in arms, legs, and joints (36, 44, 45),
- Headaches (38, 40) (42, 45).

Two studies found that nearly half of affected individuals reported three or more symptoms (35, 42).

#### 3.1.6 Comorbidity

Somatization was the most prevalent psychiatric condition comorbid with organic illnesses (25). Compared with healthy individuals, those with SSD visited physicians more frequently (34). One study noted that over one-third (41.6%) did not receive pharmacological treatment (7).

Somatization frequently co-occurred with depression and anxiety (36). In addition, patients with undifferentiated somatoform disorder showed lower scores in working memory, executive functioning, and sleep quality, and higher anxiety scores compared to healthy controls (31).

### 3.2 Illness anxiety disorder

Studies on illness anxiety originated from Egypt (n=2) (46, 47), Jordan (n=1) (48), Lebanon (n=1) (49), Saudia Arabia (n=2) (50, 51), and the UAE (n=2) (46, 52). Details of these studies and their main findings are presented in **Table 2**.

**Table 2:** Summary of studies discussing illness anxiety disorder in the Arab world.

Study	Country	Pooled sample	Scale used	Main findings
Abdel Aziz et al. (52)	UAE	Undergraduate medical students; n=193	Short Health Anxiety Inventory	-9.3% of students reached the threshold for clinically significant health anxiety. -No significant differences in characteristics were found between students with and without health anxiety.
Alsamhori et al. (48)	Jordan	Medical students; n=1,050	Self-developed questionnaire	-9.3% of participants had a high likelihood of developing illness anxiety disorder. -Among those individuals, 8% stated that fear of illness makes it difficult for them to function in society. -Students planning to visit a psychiatrist had significantly higher chances of developing illness anxiety (B=2.163, 95% CI = 1.396-3.352; *p*=0.001). -No significant differences were observed by gender or by stage of study (clinical vs. preclinical).
Bilani et al. (49)	Lebanon	Parents of children with cancer; n=105	Illness Cognition Questionnaire – Parent Version, Short Health Anxiety Inventory	-The mean parental age was 37.7 years, with most participants being married (94.3%) and mothers (78.1%). -Children mostly suffered from leukemia and had an average age of 8.4 years. -21% of parents experienced health anxiety. -Health anxiety was significantly associated with feelings of helplessness (B=0.222, p=0.021), lower acceptance (B=-0.242, p=0.008), perceived insufficient income (B=-0.238, p=0.021), and personal illness or sickness of a family member/friend (B=0.251, p=0.013).
El-Gabry et al. (46)	Egypt, UAE	Physicians; n=108	-	-34.2% of physicians reported encountering parents with illness anxiety by proxy. -33.6% reported that parents had a persistent concern that their child had a serious disease that doctors were unable to diagnose. -The most cited reason for these concerns was an exaggeration of existing symptoms (52.3%). -The concerned parent was more likely to be the mother in 48.3% of cases. -24.2% of physicians reported that most parents were unsure of their beliefs.
Ezmeirlly & Farahat (50)	Saudi Arabia	Medical students; n=271	Short Health Anxiety Inventory, the Medical Students' Disease Perception and Distress Scale	-The prevalence of illness anxiety disorder was 17%. -Younger students aged <22 years (OR=2.31, 95% CI = 1.16-4.60, p=0.02) and those with a history of a physician’s visit within the past 6 months (OR=2.46, 95% CI = 1.25-4.84, p=0.01) were more likely to have illness anxiety disorder. -The highest concern reported by one fifth of the students who visited a physician in the past 6 months was cancer (22%), followed by heart disease (8%), and infection (8%).
Hawamdeh et al. (51)	Saudi Arabia	Medical and nursing students; n=216 and 250, respectively	Short Health Anxiety Inventory, the Medical Students' Disease Perception and Distress Scale	-The prevalence of illness anxiety disorder was 38.8%. -Medical students had a significantly lower prevalence of illness anxiety compared to nursing students (57.2% *vs* 17.6%, respectively, *p*<0.001). -Fourth-year level nursing students had significantly higher scores than other nursing students (p<0.01).
Terra et al. (47)	Egypt	Medical students; n=1,173	Short Health Anxiety Inventory, 12-Item Short Form Survey	-15.7% of participants experienced clinically significant health anxiety. -A significant negative correlation was observed between health anxiety and quality of life (r=-0.393, p≤0.05). -Significant predictors of health anxiety included female gender (OR=1.41, 95% CI = 1.01-1.96, p=0.04) and lack of academic performance satisfaction (OR=1.43, 95% CI = 1.04-1.96, p=0.02).

Sample sizes ranged from 105 (49) to 1,173 participants (47). Populations included parents of children with cancer (49), physicians reporting on illness anxiety by proxy in parents (46), and medical students (47, 48, 50–52).

Reported prevalence ranged from 9.3% (52) to 38.8% (51) among medical students, and reached 21% among parents of children with cancer (49).

In medical students, health anxiety was significantly associated with lower quality of life (47), younger age (50), and a planned/recent visit to a doctor/psychiatrist (48, 50), while female gender and lack of academic satisfaction emerged as significant predictors in one study (47). Among parents of children with cancer, health anxiety was significantly associated with feelings of helplessness, lower acceptance, perceived insufficient income, and personal illness or sickness of a family member/friend (49). The most cited explanation for parental fears was an exaggeration of actual existing symptoms (46).

### 3.3 Functional neurological symptom disorder

Studies on FNSD originated from Oman (n=1) (53), Qatar (n=1) (54), and Saudi Arabia (n=2) (55, 56). Details of these studies and their main findings are presented in **Table 3**.

**Table 3:** Summary of studies discussing functional neurological symptom disorder in the Arab world.

Study	Country	Pooled sample	Scale used	Main findings
Chand et al. (53)	Oman	Children with conversion admitted to Sultan Qaboos University Hospital; n=25	–	-The most common presentation was conversion disorder with convulsions (68%), followed by conversion disorder with motor symptoms (12%), motor and sensory symptoms (8%), dissociative trance state (8%), and sensory symptoms (4%). -16% of patients had comorbid epilepsy. -8% of patients had comorbid depression.
Hamdy et al. (55)	Saudi Arabia	Patients with seizures; n=341	–	-PNES was diagnosed in 6 (1.8%) out of 341 patients. -All patients with PNES were females aged 17–27 years.
Tayeb et al. (56)	Saudi Arabia	Tertiary neuro-psychiatry referrals; n=473	–	-Out of 473 patients, 52 (11%) had functional neurological disorder. -Mean age was 34 years, and 77% were female. -Family dispute (39%) was the most reported risk factor, followed by sexual abuse (15%). -The most common symptoms were nonepileptic seizures (61.5%) and abnormal movements (30.8%). -Pain was reported by 57.7% and cognitive symptoms by 36.5%. -Symptoms were frequently attributed to supernatural causes (67.3%). -During the last follow-up visit, 53.9% reported symptom improvement, 21.2% reported no change, 10.2% reported worsening, and 15% were lost to follow-up. -The proportion of patients without symptom improvement was significantly higher among those with cognitive symptoms (p=0.018). -The mean number of visits was highest in patients reporting worsening and the lowest was among patients reporting no change (p=0.017).
Wilkins et al. (54)	Qatar	Patients admitted to the National Health Epilepsy Monitoring Unit; n=113	PHQ-9	-Of 71 confirmed diagnoses, 20 patients had PNES, 46 were diagnosed with epilepsy, and 5 had both disorders. -Individuals with PNES were significantly more likely to be native Arabs compared to those with epilepsy (p=0.003). -Patients with PNES also had more frequent seizures (p=0.051), higher mean depression scores on the PHQ-9, and more fatigue (p=0.021) -The most common referral reason in patients with PNES was recurrent/refractory seizures (p=0.011).

Sample sizes ranged from 25 children with dissociative conversion disorder (53) to 473 adult referrals to a neurology-psychiatry clinic (56). In the latter, the prevalence of FNSD was 11%, with psychogenic non-epileptic seizures (PNES) being the predominant type (56).

In studies focusing on individuals with seizures, the prevalence of PNES ranged from 1.8% (55), to 25 of 71 confirmed seizure diagnoses in an epilepsy monitoring unit in Qatar; this included 20 patients with PNES alone and 5 with both PNES and epilepsy (54).

Risk of FNSD was highest among women and patients reporting family conflict or sexual abuse (56). Compared to those with epilepsy, individuals with PNES were more likely to be native Arabs, to have more frequent seizures, and to report higher depression scores and greater fatigue (54).

### 3.4 Factitious disorder and other conditions

A total of five case reports addressed factitious disorders in the Arab world. These included one prospective case series from Oman (57) and case reports from Jordan (58), Oman (59), and Saudi Arabia (60, 61). One additional case report discussed Dhat syndrome in Oman (62). Details of these studies, including demographics, clinical presentations, and outcomes, are summarized in **Table 4**.

**Table 4:** Summary of studies discussing factitious disorder and Dhat syndrome in the Arab world.

Study	Country	Case characteristics	Main findings
Al-Habeeb (60)	Saudi Arabia	Case reports (n=2) of Münchausen in two adults admitted to a psychiatry unit	-The first case was that of a 45-year-old female with factitious dermatosis. -The second case was that of a 39-year-old man with chronic physical symptoms associated with multiple hospitalizations.
Arabi et al. (61)	Saudia Arabia	Case report of Münchausen by proxy in a male infant	-In each case, the child had recurrent episodes of severe hypoglycemia that were unexplained despite extensive investigations for endocrine and metabolic etiologies. -In each case, the child's attacks were resolved when he was briefly separated from his mother. -The diagnosis of factitious hypoglycemia imposed on another by proxy was confirmed following a scheduled change of the insulin assay in the laboratory.
Bappal et al. (59)	Oman	Case report of Münchausen by proxy in a male boy	
Bhargava et al. (57)	Oman	Case series (n=19) with long-term follow-up (two to six years) looking at the profile of Münchausen syndrome at a tertiary care hospital	-The prevalence of factitious disorder was 0.2%. -Out of the 19 patients, 42.1% had hemorrhagic factitious disorders, 15.8% feigned multiple surgical interventions, and 15.8% received a diagnosis of neurological factitious disorders. -The remaining 26.3% showed signs of minor feigned illnesses. -Objective "evidence factitia" was present in 68.4% of cases. -Following treatment, the prognosis was best for those without co-occurring psychiatric disorders
Karadsheh (58)	Jordan	Case report of Münchausen syndrome in a 16-year-old girl	-The patient was admitted to the hospital with sudden bleeding from both eyes. -Upon examination, blood was observed to pour from her eyes, but physical examination was otherwise regular. -During the patient's hospitalization, it was noted that the bleeding occurred only when the patient was alone in her room. -Later, she was observed pricking her fingertips with a hair clip and using it to cover her eyes with blood. -The patient denied having any psychological problems and did not show up for follow-up after discharge.
MacFarland et al. (62)	Oman	Case report of Dhat Syndrome in a 26-year-old male	-The patient, residing in a rural area, dropped out of university due to a distressing preoccupation with semen loss, which occurred at least once a day. -He was preoccupied with inadequacy and lack of self-esteem and doubted his masculinity. **-**The patient's blood tests were within normal limits. -Psychometric measures showed elevated scores on indices of hypochondriasis, psychasthenia, and gender role development. -Implementation of cognitive behavioral therapy, concurrent with a successful marriage proposal, resulted in a gradual resolution of the symptoms.

The case series (n=19) reported a prevalence of factitious disorder of 0.2%, with hemorrhagic presentations being the most common (57). The case reports described Münchausen syndrome in adults (60) and adolescents (58), and Münchausen by proxy in two infants (59, 61). The culture-bound Dhat syndrome case involved a young Omani male and suggested that the condition may be under-recognized in the region (62).

## 4 Discussion

In the 35 studies included in this review, SSRDs were documented across diverse Arab populations and clinical settings. Prevalence estimates for SSD ranged from 12% to 46%, while illness anxiety disorder and FNSD reached up to 38.8% and 11%, respectively, in specific subgroups. Factitious disorder and rare culture-bound presentations, such as Dhat syndrome, were reported only as isolated cases. These findings broadly align with international data; a systematic review of 32 studies from 24 countries identified a prevalence of at least one type of somatoform disorder between 26.2% and 34.8% in primary care, and up to 49% of individuals reported at least one MUS (12). European population studies likewise suggest that 8% to 20% of adults experience persistent functional symptoms, though prevalence varies by instrument and health-system context (63). Patterns of comorbidity and impairment are also consistent with global evidence: international cohorts demonstrate high rates of co-occurring anxiety and depressive disorders (64), frequent primary-care attendance, and significant work-related disability (65, 66).

Although prevalence patterns were broadly comparable to international data, the research focus within Arab studies remained narrow, emphasizing SSD over other SSRDs. Methodological heterogeneity, small sample sizes, and underrepresentation of certain countries further limited cross-study comparability. Despite these limitations, several cross-cutting themes emerged: (1) a high burden of psychiatric comorbidity, especially depression, anxiety, and trauma-related symptoms; (2) frequent co-occurrence with chronic medical illness; and (3) strong influence of sociocultural factors on symptom expression and help-seeking behavior. These patterns suggest that SSRDs in Arab contexts share core features with global presentations but are shaped by region-specific cultural norms, healthcare structures, and stigma toward mental illness.

### 4.1 Somatic symptom disorder

SSD was by far the most studied SSRD in Arab populations, with consistent associations with female gender, lower educational attainment, chronic medical conditions, and psychological distress, including depression, anxiety, and trauma-related symptoms. Research has identified a range of potential risk factors for SSD, including female gender (67), sociocultural factors (68), childhood trauma (69), genetic predisposition (70), and specific personality traits (71).

Arab refugees in host countries exhibited high rates of bodily distress, mirroring findings from refugee populations elsewhere (72, 73). This is often linked to cumulative trauma exposure, displacement stressors, socioeconomic hardship, and poor access to continuity of care (74).

Muscle and back pain were the most reported somatic symptoms, and affected individuals had comorbid mental health problems, including depression, anxiety, and trauma-related symptoms. These associations are consistent with global findings (28, 64, 69, 75) and reflect a bidirectional relationship, where psychological distress can intensify somatic symptoms, and persistent somatic symptoms can worsen psychiatric morbidity.

A particularly important finding in the Arab literature is the frequent co-occurrence of SSD with chronic organic illnesses. This complicates diagnosis and management, as clinicians must avoid both missing an underlying physical disorder and over-investigating symptoms that may have a psychogenic component (76). Furthermore, the associated history of trauma and high depression and anxiety scores reinforces the need for integrated medical–psychiatric care. Without such integration, repeated investigations and delayed recognition of underlying distress are likely to persist, increasing both patient burden and healthcare costs.

The optimal management of SSD follows a stepped-care approach grounded in the biopsychosocial model, involving close collaboration among primary-care physicians, relevant medical specialists, and mental health professionals (77). One potential framework for treatment is the CARE MD model. CARE MD is a systematic primary care management framework. It stands for Consultation, Assessment, Regular visits, Empathy, Medical/psychiatric interface, and Do no harm (78, 79). Although the evidence for this model is limited, it builds on established principles of structured care for patients with SSD/SSRD (80–82). The model emphasizes scheduled follow-up, legitimization of physical symptom distress, minimization of unnecessary investigations, empathic communication, and coordinated liaison with mental-health services as cornerstones of treatment (78, 79). In Arab contexts, these principles may be particularly relevant given the influence of stigma, supernatural explanatory models (e.g., *jinn*, evil eye), and the common practice of consulting religious or traditional healers before psychiatric services (83, 84). For example, a primary-care clinician managing a patient who attributes chronic chest pain to the evil eye might apply the CARE MD framework by scheduling regular visits to build trust and continuity, validating the patient’s suffering without endorsing supernatural causation, and engaging family members or religious and community figures, when applicable, to align biomedical and spiritual perspectives. Such culturally attuned use of CARE MD principles can reduce unnecessary investigations and enhance treatment adherence.

### 4.2 Other somatic symptoms and related disorders

Illness anxiety disorder in Arab populations was most often studied among medical students and caregivers of children with serious illnesses. Among medical students, the elevated rates of illness anxiety are consistent with the “medical student’s disease” phenomenon reported internationally (85), where increased health knowledge and academic pressure heighten health-related concerns and mental health burden (86). This suggests a need for university-based mental health programs that address both general stress management and health anxiety specifically. For parents of children with severe medical conditions, anxiety by proxy has been observed in other cultural settings (87, 88) and may contribute to increased healthcare utilization and child overmedicalization. In the Arab context, where family-centered care is highly valued, pediatric teams may need additional training to detect and address parental anxiety without undermining the therapeutic alliance.

Beyond illness anxiety, other SSRDs present distinct diagnostic and management challenges in the Arab context. FNSD, especially PNES, predominantly presented in young women and was frequently associated with trauma or family conflict. Misdiagnosis as epilepsy was common, which is consistent with global findings and has significant consequences, including unnecessary antiepileptic therapy and delayed psychiatric intervention (89–91). Limited access to diagnostic tools such as video-EEG monitoring in many Arab countries may contribute to this problem. Culturally embedded explanatory models, such as attributing seizures to supernatural causes, combined with limited expertise among healthcare providers, can further delay accurate diagnosis and hinder patient engagement in treatment (90, 92). These patterns suggest that in Arab contexts, improving access to neurodiagnostic testing and integrating culturally sensitive psychoeducation into early care pathways could reduce both diagnostic delay and inappropriate treatments.

Factitious disorders, though rare in the literature, present diagnostic and ethical challenges. Their underreporting in Arab research is likely multifactorial, reflecting stigma, medico-legal risks, and limited psychiatric service capacity for prolonged observation. Diagnostic complexity also contributes to under recognition, as differentiating factitious presentations from malingering or conversion disorders often requires multidisciplinary assessment and extended follow-up. Moreover, limited training in consultation-liaison psychiatry within the region may hinder systematic identification and reporting of such cases.

The single report of Dhat syndrome suggests that culture-bound syndromes may exist in Arab contexts but are seldom studied, possibly due to clinicians limited familiarity with such constructs. Beyond Dhat, other culture-bound syndromes have been reported in the region. For instance, *Zār* spirit possession, observed in North and East Africa and some Middle Eastern societies, manifests as dissociative episodes with behaviors such as shouting, head banging, laughing, or singing, attributed to spirit intrusion (93). Similarly, beliefs in *jinn* possession are common across Arab societies and may influence how patients interpret somatic and psychiatric symptoms (18). Such explanatory models often lead individuals to seek religious or traditional healing before psychiatric evaluation, potentially delaying diagnosis and treatment. Addressing these conditions requires culturally competent care and collaboration between mental health services and faith-based community resources.

### 4.3 Risk factors and cultural influences

Female gender emerged as a consistent risk factor for SSRDs, in line with worldwide trends (94–96). Several factors may explain this gender variation. To start with, Arab men tend to be less expressive about their illness and distress (94). This could be attributed to specific cultural norms related to the Arab world, wherein men must preserve their sense of masculinity as society defines it. Denying physical symptoms and distress can, therefore, result in men being less likely to present to the clinic with somatization. Moreover, men and women may perceive past medical experiences differently, with the former forgetting about their symptoms more readily than women (94, 97). Other potential explanations include variations in rates of anxiety and mood disorders, as well as abuse and violence between genders (98).

Cultural and religious factors influence not only who presents with SSRDs but also how symptoms are expressed. The Arab culture places a strong emphasis on the body and its sensations, while mental illness is often perceived as a sign of weakness or moral failing. Physical symptoms are, therefore, widely accepted as a legitimate expression of emotional distress. Moreover, religious beliefs and practices may influence the manifestation and interpretation of physical symptoms (99, 100). Consistent with Kleinman’s framework (101), explanatory models observed in Arab contexts often reflect culturally embedded meanings of illness, in which experiences such as *jinn* influence, the evil eye, or divine testing shape patients’ interpretation of symptoms and expectations of care (102). This cultural framing can also shape help-seeking behavior, as seen in several included studies where patients repeatedly sought biomedical consultations for physical complaints but were reluctant to accept psychiatric referral, and in the literature discussing how individuals may first consult religious or traditional healers rather than mental health professionals (103–105).

Clinicians must account for the cultural context when assessing and treating SSRDs in Arab populations. Culturally sensitive interventions, e.g., incorporating religious and spiritual components into treatment, may be effective. These interventions can include integrating religious practices, e.g., prayer, into treatment plans or utilizing culturally specific therapies, such as Hijama (cupping therapy), which is commonly used in Arab countries.

### 4.4 Implications for practice and policy

The limited availability of mental health services across the Arab region, combined with the central role of primary care in initial presentations, highlights the urgent need for clinician training in early detection and culturally sensitive communication regarding SSRDs. Primary healthcare providers should maintain a high index of suspicion for patients presenting with recurrent unexplained symptoms, to ensure early identification while minimizing unnecessary healthcare utilization. Culturally sensitive communication should be a core competency, helping clinicians explore psychological factors without alienating patients.

For SSD, patient-centered stepped care approaches that integrate the biopsychosocial approach into actual management (80–82) can reduce unnecessary investigations, while fostering trust, and improve treatment outcomes. For FNSD, particularly PNES, early diagnosis is critical to improving prognosis (106). During initial consultations, clinicians should conduct thorough mental health assessments that include screening for trauma history, psychosocial stressors, and comorbid psychiatric conditions. Physicians must also be trained in how to deliver the diagnosis clearly and empathetically. Recommended strategies include validating the patient's symptoms, naming the condition, explaining the neurobiological underpinnings (e.g., "the brain temporarily shuts down from overload"), clarifying which treatments are effective, and managing expectations (107, 108). After communicating the diagnosis, the subsequent treatment stage encompasses providing care through a multidisciplinary team that includes a neurologist, psychiatrist, psychologist, rehabilitation medicine physician, and physiotherapist (108). For illness anxiety disorder, particularly among medical students and caregivers, targeted educational interventions can help recalibrate health perceptions and reduce excessive healthcare utilization.

**Figure 2:**
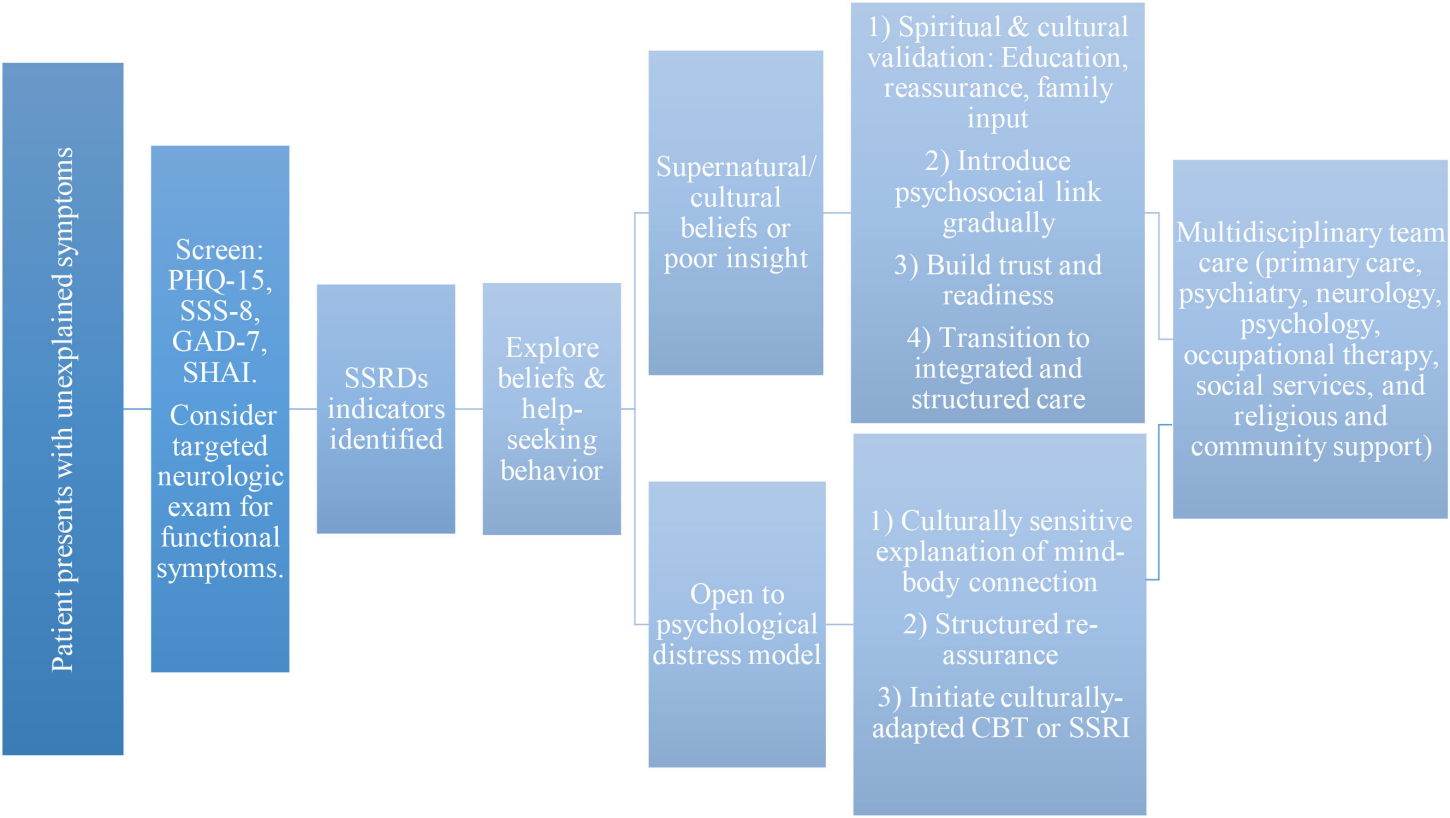
A culturally responsive clinical pathway for managing somatic symptom and related disorders in Arab primary care settings. Legend: This flowchart outlines a culturally sensitive, stepwise approach to identifying and managing somatic symptom and related disorders (SSRDs), including somatic symptom disorder, illness anxiety disorder, and functional neurological symptom disorder, in Arab populations. Initial screening is conducted using validated Arabic tools, including the Patient Health Questionnaire-15 (PHQ-15), Somatic Symptom Scale-8 (SSS-8), Generalized Anxiety Disorder-7 (GAD-7), and Short Health Anxiety Inventory (SHAI). A targeted neurological exam can be considered to further assess functional symptoms. Once SSRDs indicators are identified, the clinician explores the patient's explanatory model. For individuals who hold supernatural or culturally bound beliefs (e.g., attribution to jinn, the evil eye, fate, or curses), the care process begins with spiritual and cultural validation, through education, reassurance, and family involvement. This is followed by a gradual introduction of psychosocial explanations, aiming to build trust and prepare for transition to integrated and structured care. For patients more open to psychological models of distress, clinicians can use culturally sensitive language to explain the mind–body connection and initiate treatment such as cognitive behavioral therapy (CBT) or selective serotonin reuptake inhibitors (SSRIs). Incorporating structured reassurance that limits unnecessary investigations while validating suffering is necessary. Both pathways converge on multidisciplinary team care, including collaboration among primary care, psychiatry, neurology, psychology, occupational therapy, social services, and community-based support.

To support clinical implementation, **Figure 2** presents a culturally responsive care model tailored to Arab primary care. It integrates diagnostic pathways and screening tools that reflect culturally relevant explanatory models, aiming to enhance patient engagement, therapeutic rapport, and treatment outcomes.

At the policy level, integrating mental health services into primary care, expanding the regional mental health workforce, and developing locally relevant clinical guidelines are urgent priorities. These measures would address the current fragmentation of services, where patients often cycle through repeated biomedical consultations without psychiatric referral. Training primary care providers in culturally informed assessment and communication, drawing on evidence-based approaches such as the WHO mhGAP Intervention Guide (109, 110), can improve early recognition and reduce unnecessary investigations. Public awareness campaigns, ideally co-designed with community and religious leaders, can reduce stigma, encourage help-seeking, and normalize discussion of the mind–body connection, reframing mental health care as compatible with cultural and religious values. Policy should also incentivize research on underrepresented SSRDs and vulnerable groups, such as refugees, medical students, and rural populations, to ensure equitable service development. Investment in longitudinal and intervention studies is essential to inform sustainable, scalable models of care that reflect both the cultural context and the resource realities of the Arab region.

### 4.5 Limitations

This review has several limitations. The included studies were heterogeneous in design, diagnostic terminology, screening tools, and populations, limiting direct comparison. Most employed cross-sectional designs and self-report measures, reducing diagnostic precision. Small sample sizes and reliance on convenience sampling limited generalizability. Few or no studies examined longitudinal outcomes, cultural explanatory models, or the healthcare system impact of SSRDs. Given these limitations, the review should be interpreted as a broad synthesis rather than a definitive measure of prevalence or causality. Future work should employ validated DSM-5-TR–aligned tools, investigate gender-specific and culturally informed pathways, and assess the effectiveness of culturally adapted interventions in routine care.

## 5 Conclusion

SSRDs are prevalent in Arab populations, with SSD most frequently studied and marked by high psychiatric comorbidity, overlap with chronic medical illness, and culturally shaped help-seeking patterns. Gender differences and stigma influence symptom expression and delay psychiatric referral. Improving outcomes will require early recognition in primary care, culturally sensitive communication, and patient-centered stepped care approaches. Future research should explore longitudinal outcomes, trauma pathways, and the effectiveness of culturally adapted interventions to guide locally relevant policy and clinical guidelines.
